# Toddlers’ symbolic play in dyadic mother–child and triadic mother–child-older-sibling interactions

**DOI:** 10.3389/fpsyg.2026.1858906

**Published:** 2026-08-25

**Authors:** Sharon Sherman, Naomi Havron

**Affiliations:** 1 School of Psychological Sciences, University of Haifa, Haifa, Israel; 2 The Institute for Information Processing and Decision Making, University of Haifa, Haifa, Israel; 3 The Center for the Study of Child Development, University of Haifa, Haifa, Israel

**Keywords:** dyadic interactions, play, siblings, symbolic play, triadic interactions

## Abstract

Symbolic play supports crucial aspects of early childhood development, including language and social-cognitive skills. Parent-child dyadic play has been shown to facilitate symbolic play development, but less is known about the addition of siblings (triadic interactions), which may present both opportunities and challenges. We examined toddlers' symbolic play across dyadic (mother-child) and triadic (mother-child-sibling) free-play interactions recorded through videoconferencing. Twenty toddlers (aged 1.5–2.5 years, eight girls) were observed playing with their mothers and older siblings (aged 3.5–7.5 years, ten sisters) in two standardized 10-minute play sessions at their homes. We coded the frequency, duration, variety, toddler initiative, and complexity of symbolic play. Consistent with our hypothesis, toddlers demonstrated significantly higher levels of symbolic play in dyadic versus triadic interactions overall (*p* < 0.0001, Cohen's *d* = 0.953) and across all measured dimensions (*p* < 0.05 to *p* < 0.001, Cohen's *d* = 0.637–0.915). This suggests that dyadic play may optimize conditions for symbolic play development, and/or that the addition of siblings introduces competing demands that reduce opportunities for advanced symbolic play. The results have implications for understanding how family configuration influences early play experiences and may inform the design of interventions to support symbolic play development in different social contexts.

## Introduction

Symbolic play is a significant component of children’s development (Quinn et al., 2018). In typically developing children, symbolic abilities increase significantly between the second and third years of life, primarily expressed through language development and symbolic activities in play (Lyytinen et al., 1997; Piaget, 1962; Ungerer and Sigman, 1981). This type of play reflects children’s capacity for representational thought, including symbolic representation of objects, events, and people, and the ability to differentiate between these symbols and their meanings (Piaget, 1962; Vygotsky, 1967). Research has demonstrated that symbolic play benefits children’s cognitive development (Kowalski et al., 2004), language learning, social relationships (Casby, 2003; Erikson, 1951), perspective-taking, and emotional development (Christensen et al., 2010; Colwell and Lindsey, 2003; Fein and Fryer, 1995). For example, Creaghe et al. (2021) found that symbolic play elicits more conversational turn-taking and richer infant-directed speech than functional play.

The ability to play symbolically requires cognitive abilities that develop gradually during children’s early years, progressing from simple symbolic play in infancy to more complex play later on (Baron-Cohen, 1987; Ungerer and Sigman, 1981). Initially, infants exhibit sensorimotor play, involving simple manipulation of objects (e.g., stacking and throwing them) or focus on objects’ physical properties (Christensen et al., 2010; Ungerer and Sigman, 1981). Around one year of age, children begin to demonstrate functional play, using objects for their intended purpose, such as pushing a toy car or placing a toy cup on a saucer (Christensen et al., 2010; Ungerer and Sigman, 1981). By age two, supported by developing cognitive abilities for representing and mentally transforming objects (Leslie, 1987; Lyytinen et al., 1997), symbolic play emerges. Children can then use one object to represent another or create imaginary objects (e.g., feeding a doll or using a block as a phone; Ungerer and Sigman, 1981). Symbolic play develops both quantitatively and qualitatively with age, as children demonstrate increased play duration and engage in more sophisticated and planned play (Linder, 1993; Rutherford and Rogers, 2003; Slade, 1987). Integrating this progression with the development of manual skill, Lockman and Tamis-LeMonda (2021) argue that children’s real and pretend uses of objects are reciprocal and mutually scaffolding, such that advances in one domain support advances in the other.

## Symbolic play with a parent

The development of symbolic abilities is influenced by both biological and environmental factors, including children’s early social relationships and their play partners (Fein and Fryer, 1995; Rutherford and Rogers, 2003; Slade, 1987). Play partners’ characteristics—such as their developmental level and motivation—are related to the development of symbolic ability (Slade, 1987). As parents are central figures in a child’s immediate environment and development (Bronfenbrenner, 1977), they play a crucial role in play development. Around age two, when children begin to engage in symbolic play, parental involvement becomes critical (Fiese, 1990; Slade, 1987). Parents enable toddlers to reach the Zone of Proximal Development, where they can perform actions with assistance but cannot yet perform them alone (Marjanovič-Umek et al., 2014; Slade, 1987; Vygotsky, 1987). Consistent with this view, Creaghe and Kidd (2022) showed that the informational exchange between caregiver and child during symbolic play reflects such a zone of proximal development, as the ambiguity inherent in pretense prompts the pair to jointly negotiate shared meaning. Parent–child dyadic play exposes the child to content and opportunities that encourage higher-level symbolic play, increases their motivation to practice symbolism, and enhances their representational and symbolic abilities (Fein and Fryer, 1995; Slade, 1987). Joint play interactions with mothers are associated with increases in both the quantity and duration of symbolic play episodes, as well as qualitative improvements in the diversity, sophistication, and complexity of these episodes, compared to children playing alone or with other children (Dunn et al., 1977; Fein and Fryer, 1995; Fiese, 1990). Observing naturalistic play at home, Fletcher et al. (2020) found that the frequency of mothers’ symbolic play was closely tied to their toddlers’ own symbolic play across the second and third years of life.

The positive influence of mother–child joint play depends on various factors. To sustain play and maintain the child’s interest and engagement, maternal initiatives, active involvement, and facilitation of reciprocity and turn-taking are essential (Fein and Fryer, 1995; Fiese, 1990; Slade, 1987). The sophistication of symbolic play is contingent upon maternal guidance, verbal comments, and the mother’s modelling in play (Marjanovič-Umek et al., 2014; O'connell and Bretherton, 1984; Slade, 1987). Demonstrating this experimentally, Stuart et al. (2023) found that introducing a structured story stem into mother–child play raised toddlers’ developmental play level and lengthened the duration of their pretend play relative to free play. Conversely, Rao et al. (2021) found that mothers reporting higher anxiety engaged in less pretend play with their toddlers, and that mothers’ engagement in pretend play predicted fewer child behavioural problems two years later, underscoring the developmental significance of mothers’ participation in play.

For the benefits of maternal play to occur, precise alignment with the child’s play level is necessary (Palacios and Rodríguez, 2015). This alignment involves adaptation and adjustment that both challenge the child’s symbolic abilities and maintain sufficient balance to promote positive outcomes in dyadic play (Marjanovič-Umek et al., 2014). Consequently, not every play partner may equally benefit the child’s play development.

Research supporting these claims demonstrates a birth-order effect on children’s symbolic play, with younger siblings exhibiting reduced and lower-level symbolic play compared to older siblings (Kowalski, 2000; Kowalski et al., 2004; Lyytinen et al., 1997). One proposed explanation is that older siblings receive more joint symbolic playtime with their parents than younger siblings do (Kowalski, 2000; Kowalski et al., 2004). Moreover, younger siblings may experience more external distractions during play and receive less exclusive parental attention compared to their older siblings (Kowalski et al., 2004).

## Symbolic play with siblings

The sibling relationship is often the longest-lasting relationship in a person’s life and represents a central relationship in children’s early development. Within sibling relationships and interactions, significant social-cognitive development and learning occurs (Bronfenbrenner, 1999; Kowalski et al., 2004). Similar to symbolic play with parents, play with an older sibling who typically possesses higher symbolic-play skills may catalyze the toddler’s symbolic play development (Kowalski, 2000).

Observational research by Dunn and Dale (1984) indicates that in shared symbolic play between siblings, older siblings typically establish the roles, identities, positions, and psychological states in play before inviting the toddler sibling to join. This provides support and a foundation for higher-level symbolic play. Beyond the modeling and guidance provided by the older sibling, the proximity in age and content familiarity also supports learning and development during play (Kowalski, 2000; Kowalski et al., 2004). Studies have found that during joint play with an older sibling or peer, toddlers engage in more complex symbolic play with higher frequency compared to solitary play or play with same-age peers (Kowalski, 2000; Kowalski et al., 2004; Youngblade and Dunn, 1995).

Despite the potential benefits of sibling interactions for play development, such play may not provide the same advantages as parent–child play. Research suggests that parental presence encourages more mature, instructive, and diverse play in toddlers compared to play with older siblings, providing a higher-level verbal and intellectual environment (Stevenson et al., 1988; Teti et al., 1988). While parent–child play is characterized by encouragement, verbal guidance, and scaffolding, sibling play often involves more parallel play (Stevenson et al., 1988; Teti et al., 1988).

Furthermore, older siblings tend to take on a more controlling, dominant, and directive role in play with their younger siblings, mainly exploring the functional properties of toys while the toddler observes (Stevenson et al., 1988). Moreover, in sibling symbolic play, there is less adaptation between partners compared to parent–child play. This adaptation is known to play an important role in the development of symbolic play (Stevenson et al., 1988; Teti et al., 1988).

Beyond the potentially lower contribution of sibling play compared to parent play, in some cases, play with siblings may also have negative effects. Sibling relationships may contain negative elements such as conflict and competition, which can penetrate siblings’ shared play and affect its quality (Baskett and Johnson, 1982; Oden and Hall, 2021). Some sibling characteristics can influence their relationships and their shared play, such as gender, birth order, and the siblings’ ages (Dunifon et al., 2017; Oden and Hall, 2021). For example, older sisters may be more positive in play with toddlers compared to older brothers (Oden and Hall, 2021), and were also found to have a differential effect on language outcomes, such that having an older brother was associated with lower language outcomes than having an older sister (Havron et al., 2019). An interaction between age and gender may also be related to language development, whereby siblings of a certain age contribute similarly to caregivers, but sisters are considered as “caregivers” from an earlier age than brothers (Rosslund et al., 2025). However, in the field of language development, a smaller age gap has also been found to be related to better language outcomes than a larger age gap (Havron et al., 2022; Gurgand et al., 2023).

## Triadic symbolic play

Since the majority of children live with a sibling (over 80% in the United States) and often spend most of the hours they are home engaged in play with their siblings (Dunifon et al., 2017), they tend to participate in family interactions rather than necessarily in dyadic interactions with the parent alone (Dunifon et al., 2017; Rogoff, 2003). A recent study conducted in Israel on families with two children found that triadic or family interactions were about five to ten times more common than any other interaction configuration (Netovich, 2024). Therefore, examining toddlers’ play within a triadic framework (toddler-parent-sibling) is of high importance.

There is evidence that the play behavior of mothers with toddlers differs significantly from their play behavior with their older children (Stevenson et al., 1988). This may make it difficult for the mother to adapt her play to both of her children simultaneously in a way that benefits each of them during triadic play. The few studies that have examined triadic interactions have found that these interactions affected the mother’s ability to engage in joint attention with her toddler and to provide responsive assistance, and that the quality of the interaction decreased depending on the number of people involved (Benigno et al., 2007; Benigno and Ellis, 2004; Oshima-Takane and Robbins, 2003).

Furthermore, it has been found that in triadic interactions, younger siblings tend to show less active participation compared to dyadic interactions, older siblings are more active partners than younger ones, and more negative emotions and conflicts appear (Finn and Vandermaas-Peeler, 2013). Additionally, the presence of an older sibling in the play interaction negatively impacted the parent’s teaching and guidance for the toddler, and the interaction tended to be more tailored to the level of the older sibling (Benigno and Ellis, 2004; Benigno et al., 2007).

Consistent with these findings, Oshima-Takane and Robbins (2003) found that, in terms of maternal linguistic input, the presence of an older sibling in shared play altered the interaction between the toddler and the mother, creating a linguistic environment less conducive to learning. In triadic interactions, the mother speaks less to the toddler compared to dyadic conditions, directs her speech more toward the older sibling than the younger one, and the older siblings respond to their mother before their younger siblings can do so. It is reasonable to expect that similar patterns will occur in the context of symbolic play, such that the mother’s play actions in triadic interactions will tend to be directed more toward the older sibling.

Providing feedback that strengthens symbolic understanding contributes to the quality of play but requires attention to the toddler—which may be negatively affected by the presence of an additional child in the play activity (Palacios and Rodríguez, 2015). Furthermore, it has been suggested that the difficulty resulting from the presence of the older sibling in the interaction occurs because, in a triadic framework, parents are more focused on mediation, negotiation, conflict prevention, and ensuring that their children collaborate, compared to dyadic frameworks (Benigno and Ellis, 2004; Oshima-Takane and Robbins, 2003).

A theory that may explain this is the resource dilution theory, which argues that parental resources are limited, so as the number of children in a family increases, the parental resources provided to each child decrease (Blake, 1981; Downey, 2001). A reduction in important parental resources for a child’s development may thus affect their symbolic play. Findings indicating lower symbolic-play skills in younger siblings compared to older siblings (Kowalski et al., 2004; Lyytinen et al., 1997) support this argument.

However, in contrast to these claims, Barton and Tomasello (1991) demonstrated that conversations between mothers and their children in triadic interactions were three times longer than conversations in dyads with each child, concluding that toddlers experience a richer learning environment when their older siblings are present. Furthermore, according to Woollett (1986), although the presence of an older sibling may lead to a less sensitive environment for the younger sibling, it also creates a more stimulating environment with more complex models for the toddler’s development, potentially aiding in their development. In the field of language acquisition, Loukatou et al. (2022) found that overheard speech directed to another child is similar in its beneficial characteristics to speech directed to the target child (usually referred to as Child-Directed Speech). This could mean that, in a triadic interaction with a parent and sibling, a child would be attentive to speech directed at the older sibling, benefiting more than if this was overheard speech directed to another adult.

Since existing findings on the advantages and disadvantages of triadic interactions for toddlers are mixed, further research is needed to examine the effects of older siblings on the interaction between toddlers and their mothers. Moreover, there is almost no research on triadic play interactions of toddler-mother-sibling in comparison to dyadic interactions, despite children learning from their siblings and engaging in shared activities with them and their parents on a daily basis (Finn and Vandermaas-Peeler, 2013; Rogoff, 2003).

## The current study

As mentioned, there are many benefits to a toddler’s symbolic play with their mother (Kowalski, 2000; Slade, 1987), but other key partners in the toddler’s play in their early years are their older siblings, who also influence their symbolic play. Findings regarding the influence of older siblings on the development of symbolic play are equivocal (Kowalski, 2000; Kowalski et al., 2004; Youngblade and Dunn, 1995). Since there is almost no research on symbolic play in triadic interactions and its implications, the focus of the current research is on examining the influence of the involvement of an older sibling in toddlers’ play interactions with their mothers on toddlers’ symbolic play. Due to the known benefits of joint play between toddlers and their mothers, the dyadic symbolic play interaction of the toddler with their mother alone is compared to a triadic symbolic play interaction including the toddler, their mother, and their older sibling. Unlike previous research, where the comparison between dyadic and triadic play was made between different groups of participants (e.g., Finn and Vandermaas-Peeler, 2013), in the current study, the toddler participated in two play conditions that were identical except for the presence of their older sibling, allowing for a direct assessment of the influence of the older sibling’s presence on that same toddler.

Toddlers were in the middle to end of their second to third year of life. This age range allows for an examination of symbolic abilities that already exist among them (Kowalski, 2000; Lyytinen et al., 1997; Ungerer and Sigman, 1981), and it also enables younger siblings to participate in triadic interactions (Barton and Tomasello, 1991). The study includes only mothers and not fathers (Benigno et al., 2007; Farver and Wimbarti, 1995; Tomasello, 1991), since mothers tend to be the primary caregivers in Israel, and there may be differences in play between fathers and mothers that complicate the comparison between them (Keren et al., 2005).

The aim of the study is to examine differences between the symbolic play of the toddler in a dyadic play interaction with only their mother and a triadic play interaction including their sibling. We hypothesize that measures of symbolic play, including frequency, duration, variety, child initiative, and complexity, will be higher in the dyadic play condition compared to the triadic play condition.

## Method

### Participants

Data collection began in July 2023 and was paused in October 2023, earlier than initially planned, owing to geopolitical events that made continued testing impossible. Twenty triads participated in the study, recruited through social media. The triads included toddlers aged 1.5–2.5 years (*M* = 25.2 months, *SD* = 3.403, 8 girls and 12 boys), their older siblings aged 3.5–7.5 years (*M* = 65.75 months, *SD* = 13.75, 10 girls and 10 boys), and their mothers. The mean age difference between the toddlers and their older siblings was 40.56 months (3.38 years; S*D* = 13.92 months, 1.16 years), ranging from 1.30 to 5.67 years. All participating children were born full-term and were typically developing. The participants were monolingual Hebrew speakers, except for one child who was bilingual. Additionally, one of the participants had a twin diagnosed with Autism Spectrum Condition (who did not participate in the study). Thirteen of the toddlers had no additional siblings apart from those participating in the study; the others had additional siblings. Half of the mothers held a bachelor’s degree (*n* = 10) and half held a master’s degree or higher (*n* = 10). Fathers’ highest reported level of education was a bachelor’s degree (*n* = 12), a master’s degree or higher (*n* = 4), a medical degree (*n* = 1), vocational training (*n* = 2), and a high-school diploma (*n* = 1). Mothers reported being the primary caregiver for their children in 9 families, while in the remaining 11 families caregiving was reported to be shared equally between the two parents. Nineteen mothers identified the family as Jewish and one as other. Reported combined gross monthly household income (across both parents) averaged approximately 36,900 ILS (S*D* = 15,900; range 16,000–80,000; median 35,000) among the 17 families who provided this information.

### Procedure

**Figure 1:**
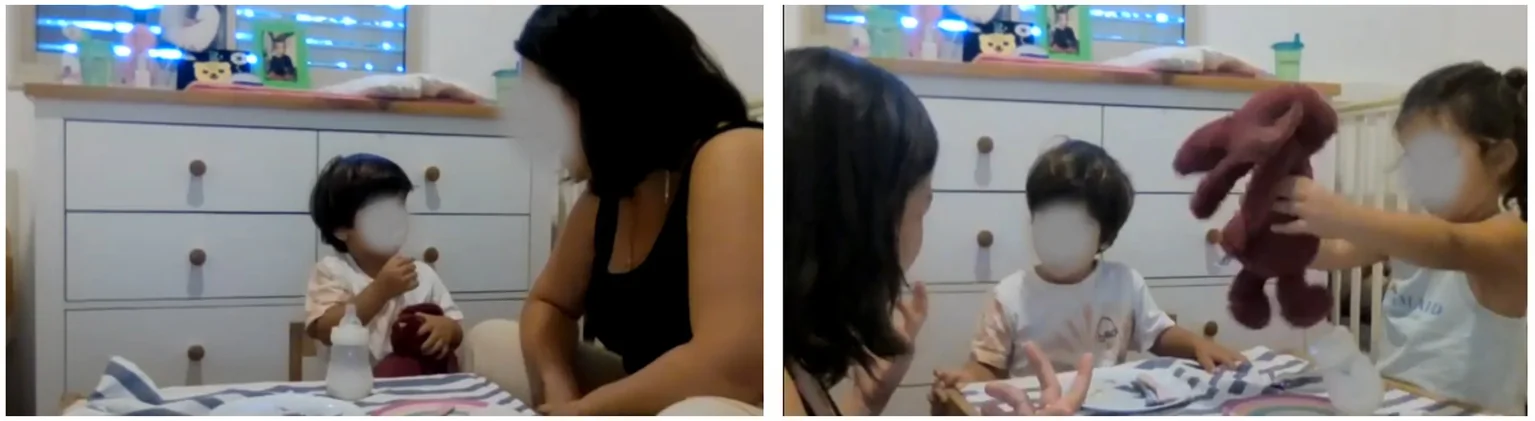
An example of the study setup. On the left, a dyadic interaction, on the right, a triadic interaction.

The study consisted of one video-conferencing session from the participants’ home, where video recordings of the toddlers’ play interactions were recorded under two different conditions: a dyadic play interaction condition lasting 10 min, where the toddler and mother engaged in free play with toys; and a triadic play condition, where the toddler played with their older sibling and mother simultaneously, also in free play lasting approximately 10 min (see Figure 1). Mothers were told beforehand to arrange for the second child to be occupied while the dyadic condition was recorded. During the play sessions, the experimenter turned off her video so that the participants would not be distracted by her. She did, however, remain present, told the mother when the ten minutes had passed, and monitored the transition between conditions. The order of conditions was balanced across participants by creating an excel sheet with a randomized order before recruitment began, and only correcting the order toward the end of testing to create equal groups. In both conditions, the children used a set of toys from a predefined list that encouraged symbolic play (see Supplementary Appendix A) and were instructed to play in a fun way, as they usually would. The procedure was based on Marcu et al. (2009), except that we decided to allow a larger set of toys to promote more natural play and allow for personal preferences, and that each play episode lasted 10 rather than 8 min. A standardized procedure was used to manage this transition. Before the session, mothers were told that they would need to add or remove the older sibling between conditions, and were asked to do so as smoothly and pleasantly as possible. At the start of the session, the children were also prepared in advance and reminded that, for part of the play, the older sibling would not take part or they would join in. At the end of the first ten-minute condition, the experimenter announced the change, stating that the time for that part was over and that the older sibling would now either join the play or leave for a few minutes so that only the mother and the toddler would remain (depending on condition order), and she then verified that the change had taken place. Beyond announcing and confirming the transition, the experimenter did not direct how it was carried out; mothers managed the practical change with their children using whatever strategies best suited them and their families. Timing of the second condition began only once the transition had been fully completed and the dyad or triad had re-settled into play.

Data were collected remotely via video conferencing rather than in a laboratory in order to observe toddlers in their familiar home environment, playing with their own toys, while keeping the procedure standardized across families. Conducting the sessions at home, and turning off the experimenter’s video during play, was intended to reduce the unnaturalness and reactivity associated with a laboratory visit and an observer’s visible presence, as well as make recruitment easier.

All parents signed an informed consent form and the study was approved by the [removed for review] ethics committee. Families were recruited through social media. As appreciation for their participation, families received a 50 ILS voucher to purchase books.

### Measures

#### Assessment of the child’s symbolic play level

The assessment of the child’s symbolic-play level was conducted by coding video observations of dyadic and triadic play interactions. The observations allowed for an assessment of the direct influence of the older sibling on the toddler’s symbolic play, using measures independent of parental reports. The coding process was based on tools developed by Marcu et al. (2009) for evaluating children’s symbolic play level and additionally elaborated or adapted by the authors (see Supplementary Appendix B).

The measures coded to evaluate the toddler’s symbolic play level were:

1. Duration of symbolic play: The total duration of symbolic play in the interaction in seconds.
2. Frequency of symbolic play: The number of segments of symbolic play during the interaction.
3. Variety of symbolic play: The number of different types of symbolic play actions performed (e.g., feeding a teddy bear and putting a teddy bear to sleep were counted as two different actions).
4. Toddler initiative in symbolic play: The number of times the toddler was the one who initiated symbolic play segments.
5. Maximal complexity of symbolic play: The highest symbolic complexity level reached by the toddler in each play interaction, ranging from 1 to 5 (see Supplementary Appendix B).

These measures allowed for an assessment of symbolic play both quantitatively (duration, frequency) and qualitatively (complexity, variety), with child initiative not easily fitting into any of these categories.

#### Coding

All segments of play were first identified from the observation, defined as segments starting from an action (physical or verbal) on an object, or from the moment of contact with it until its end. For an action of the child to be considered as part of play, the child had to pay attention to the object. Additionally, symbolic play was differentiated from non-symbolic play. Symbolic play was defined as play involving the use of one object to represent another, attribution of invisible properties to an object, reference to nonexistent objects, or treating an object as an agent (Marcu et al., 2009; Slade, 1987; Ungerer and Sigman, 1981).

To assess the reliability of the coding, 20% of the videos, which are 8 videos from 4 children, were coded by an additional coder. Categorical agreement between the two coders was evaluated at the level of individual play segments. Agreement on segmentation itself, that is, whether the two coders identified play in the same portions of the session, was assessed second by second across the recordings: coders agreed on 82.9% of one-second intervals, rising to 89.2% when boundaries falling within one second of each other were treated as agreeing.

For the classification of a segment as symbolic versus non-symbolic (260 matched segments), we found a Cohen’s *κ* = 0.92, and prevalence-and-bias-adjusted kappa (PABAK) = 0.92. Among segments both coders classified as symbolic (*n* = 137), for the questions of whether a new symbolic theme was introduced we found a *κ* = 0.76, PABAK = 0.77; for whether the play was child-initiated *κ* = 0.70, PABAK = 0.71, and for the ordinal complexity rating linearly weighted *κ* = 0.65, PABAK = 0.66. These values suggest that some classification decisions were harder than others. There was large agreement on the most basic decision, of whether a segment was symbolic, but whether a symbolic play segment represented the same or different theme was more subjective. For example, one segment where a child fed himself was coded as a new theme by one coder, and as a continuation of a previous theme (feeding the bunny) by the other. Child-initiation was likewise more subjective than whether the segment was symbolic, for example, in one segment the mother made a suggestion that the child blow on the food to make it colder, but the child chose to feed the rabbit – one coder considered this to be child initiated and one did not. Finally, complexity seems to be the less reliable measure, as it includes multiple possible levels, and required making high level decisions about continuity. For example, in one disagreement, one coder saw the segment as a simple symbolic play, while the other saw the segment as part of an elaborate sequence rather than a simple act. In cases of coding disagreement, the coders held a discussion, and a consensus was reached. The final coding served as the basis for data analysis. The recordings were coded using ELAN (Wittenburg et al., 2006).

#### Demographic questionnaire

Participants were asked about their children’s age, gender, educational settings, concerns about health or developmental issues, hearing or vision problems, parental education, languages spoken at home, number of children in the household, and their birth order and gender.

Parents also completed a short symbolic-play questionnaire (MB-CDI subscales), which showed no significant associations with the observational measures and was not analyzed further (see Supplementary Appendix D).

### Data analysis

While the study was not formally preregistered, the research proposal was submitted and approved by the MA committee of the [removed for review] on March 2023. The original research proposal and a corresponding English translation are available on the OSF directory. All statistical analyses were conducted in R (R Core Team, 2020), using *effsize* for effect sizes (Torchiano, 2020), *irr* for inter-rater reliability (Gamer et al., 2019), *pwr* for power analyses (Champely, 2020), DescTools (Signorell, 2025) and *ggplot2* with *patchwork* for figures (Wickham, 2016; Pedersen, 2025).

The initial analysis plan was to examine the measures of symbolic play using variables expressed as proportions (for example, variety calculated by the number of different types of play actions out of the total symbolic play segments). However, during the coding process, it became apparent that proportion variables tended to exhibit bias in cases where there was little play in one of the conditions (usually the triadic one), leading to significant weight given to each play segment. For example, if a child only played one new play theme in the triadic condition, they would have 1 new action out of 1, and the score for variety would be 1. If the same child had 10 play actions in the dyadic condition, with only five of them new, they would then have a lower score of 0.5.

To examine this empirically, we examined the correlations between the proportion variables and the variables expressed in absolute values. Proportion variables were not consistently correlated with absolute variables and were sometimes even negatively correlated. Consequently, it was decided to examine dependent variables that are not expressed as proportions but as absolute numbers (see Supplementary Appendix C for details on analysis using the proportion variables: results are similar to those reported below).

To examine the research hypothesis, we conducted an omnibus multivariate test of the condition effect using a one-sample Hotelling’s T^2^, the multivariate analogue of the paired-samples t-test. For each participant we computed a vector of within-subject difference scores (dyadic − triadic) across the play measures and tested the null hypothesis that the vector of mean differences equalled zero. Because two toddlers exhibited no symbolic play in the triadic condition, leaving maximal complexity and child initiative undefined for them, the test was run at two scopes: across all five measures for the 18 children with complete data, and then across the three measures available for the full sample (duration, frequency, and variety; *N* = 20). Next, a series of *t*-tests was conducted for each dependent measure separately. Because there was no effect for condition order on any of the variables (*t*-values ranged from 1.809 for ‘duration’ to 0.489 for ‘complexity’), we collapsed the two orders. Prior to the paired-samples comparisons, the distribution of the difference scores (dyadic minus triadic) was examined for each measure using the Shapiro–Wilk test. The difference scores were consistent with normality for the duration, frequency, and variety of symbolic play and for child initiative (all *p* > 0.20), but not for maximal complexity (W = 0.77, *p* = 0.001), reflecting the restricted range of the complexity scores. The paired t test for complexity was therefore corroborated with a non-parametric Wilcoxon signed-rank test. To address the risk of alpha inflation arising from testing the five symbolic-play measures, the *p* values were adjusted using the Holm–Bonferroni procedure.

With the present sample (*N* = 20; *N* = 18 for complexity and child initiative, which could not be computed for the two toddlers who displayed no symbolic play in the triadic condition), achieved power was high for the large effects observed for duration, frequency, variety, and initiative (> 0.97 at *α* = 0.05 and > 0.88 at the Bonferroni-adjusted *α* = 0.01), but lower for the smaller complexity effect (dz = 0.52; power = 0.55 at *α* = 0.05). A sensitivity analysis indicated that the design afforded 80% power to detect within-subject effects of dz. ≈ 0.66 (*N* = 20) and dz. ≈ 0.70 (*N* = 18).

## Results

Data and analysis scripts are available on the OSF link: https://osf.io/rqs8w/?view_only=8ea622ebae3b49c0849a2fd2e316c419.

### Descriptive statistics

Table 1 presents the means, standard deviations, and ranges of background variables and dependent variables examined in the current study.

**Table 1:** Means, standard deviations, and ranges of background variables and dependent variables.

	Mean (SD)	Range
Toddler’s age (months)	25.193 (3.403)	19.2–30.6
Toddler’s gender	40% girls	
Older sibling age (months)	65.752 (13.748)	42.1–89.6
Maternal education	50% Bachelor’s degree, 50% Master’s degree and above	
Overall duration of symbolic play segments (seconds)	86.728 (62.95)	0–274.88
Overall duration of all play segments (seconds)	189.151 (69.52)	34.7–329.7
Proportion of symbolic play out of overall play	0.451 (0.23)	0–0.923
Number of symbolic play segments	18.15 (13.68)	0–52
Number of all play segments	33.275 (14.14)	5–70
Proportion of symbolic play segments out of all play segments	0.502 (0.25)	0.04–0.95
Variety (number of new symbolic themes)	9.675 (7.25)	0–29
Proportion of new symbolic themes out of the overall number of symbolic segments	0.586 (0.18)	0.26–1
Toddler initiative (the number of toddler-initiated symbolic play segments)	11.158 (8.35)	1–32
The proportion of toddler-initiated symbolic play segments out of all symbolic play segments	0.603 (0.21)	0.17–1
Maximal complexity of symbolic play (scale of 1–5)	3.579 (0.68)	1–4

**Figure 2:**
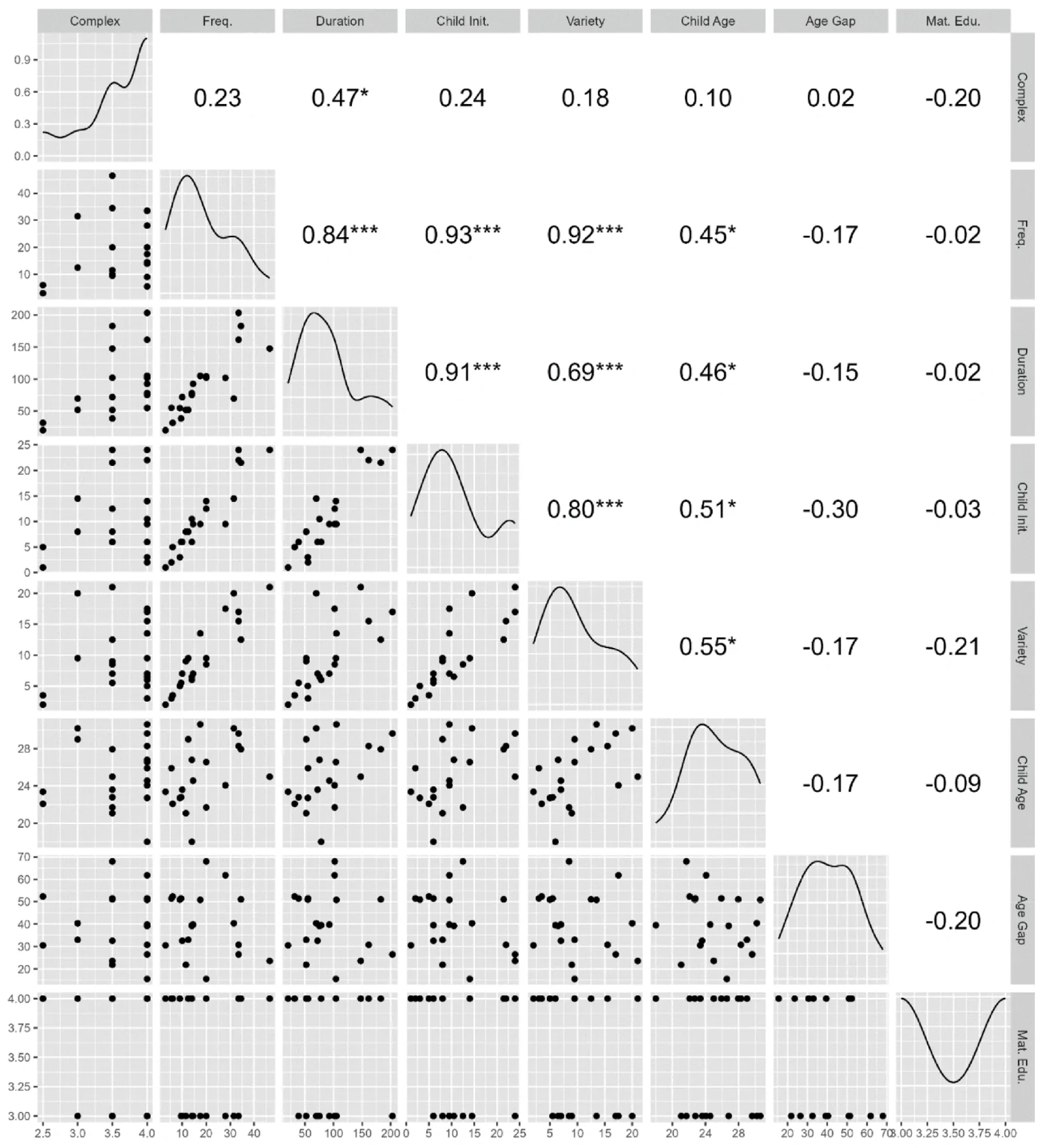
Correlations between all dependent and independent variables.

As an exploratory, descriptive examination of how the play measures related to one another and to background variables, we inspected the correlations shown in Figure 2. These analyses were not hypothesis-driven and, given the small sample and the large number of pairwise tests involved, are statistically underpowered and uncorrected for multiple comparisons; we therefore describe the overall patterns rather than interpreting individual coefficients or their significance, and they should be read as hypothesis-generating only. Descriptively, the age of the toddler was correlated with frequency, duration, variety, and initiative, but not complexity—such that as the toddler’s age increased, the symbolic play measures (except complexity) were higher. No correlations were found for the age of the siblings, maternal education, or order of conditions for any of the dependent variables. All dependent variables had significant correlations with each other, except for the variable ‘complexity,’ which was found to have a significant correlation only with ‘duration’ (see Figure 2).

### Hypothesis testing

Two children did not perform any symbolic play in the triadic condition and were removed from analyses looking at complexity, child initiative, and overall symbolic play score. Revealingly, both children did show symbolic play in the dyadic condition. These two children were excluded from the complexity and child-initiative analyses for a definitional reason: with no symbolic play in the triadic condition, the maximal complexity and the number of child-initiated symbolic acts in that condition are undefined rather than zero. They were retained in the duration, frequency, and variety analyses, where the absence of symbolic play is meaningfully scored as zero (hence *N* = 20 for those measures and *N* = 18 for complexity and initiative).

The multivariate Hotelling’s T^2^ examining the main research hypothesis was significant both for the three measures available for all participants [*N* = 20; Hotelling’s T^2^ = 33.21, *F*(3, 17) = 9.91, *p* < 0.001] and for all five measures among children with complete data [*N* = 18; Hotelling’s T^2^ = 55.23, *F*(5, 13) = 8.45, *p* < 0.001], with all mean differences favoring the dyadic condition.

**Figure 3:**
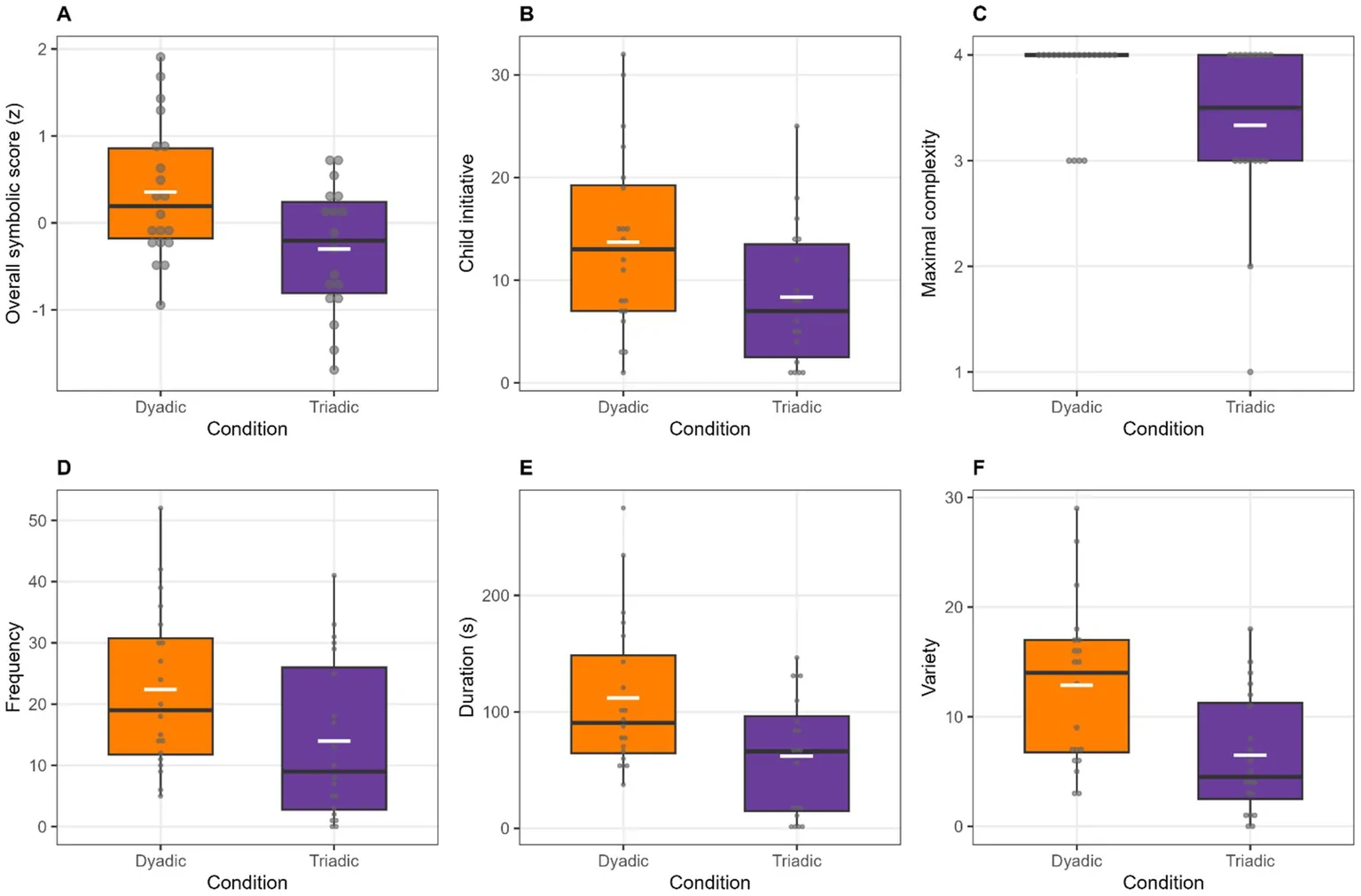
Plots for overall symbolic score and each dependent measure of symbolic play in the two conditions. Dyadic condition in orange and triadic condition in purple. **(A)** Overall symbolic score (in Z scores). **(B)** Child initiative, **(C)** Maximal complexity, **(D)** Number of symbolic play segments (frequency), **(E)** Duration, **(F)** Variety. The grey dots represent individual participants. The lower and upper hinges correspond to the first and third quartiles, the dotted white lines represent the means, and the black lines within the squares represent the median. The top whiskers denote the maximum value, and the bottom whiskers the minimum value.

All symbolic play measures were significantly influenced by the type of interaction. Thus, the duration of symbolic play, frequency of symbolic play, complexity of symbolic play, variety of symbolic play, and initiative of symbolic play were all higher in the dyadic play of the toddler and their mother compared to the triadic play with the mother and sibling (see Table 2 and Figure 3). As a single interpretable summary of the condition effect and for visualization (Figure 3A), we averaged the five standardized (z-scored) measures into an overall symbolic-play score. This composite symbolic score was significantly higher in the dyadic interaction (M = 0.428, S*D* = 0.791) than in the triadic interaction [M = −0.303, S*D* = 0.738, t(17) = 5.494, *p* < 0.0001, 95% CI (0.450, 1.012), *d* = 0.953].

**Table 2:** Means, standard deviations, *t*-values, df, *p*-values, 95% CIs, and Cohen’s d for the five dependent measures.

	Mean (SD) Dyadic	Mean (SD) Triadic	*t*	df	*p*	Cohen’s d	95%CIs	Holm–Bonferroni corrected threshold
Variety	12.85 (7.52)	6.50 (5.51)	5.266	19	<0.0001	0.915	0.496, 1.335	0.01
Duration	111.71 (65.14)	61.75 (50.79)	4.448	19	<0.001	0.832	0393, 1.272	0.0125
Frequency	22.35 (13.25)	13.95 (13.11)	4.199	19	<0.001	0.637	0.3, 0.974	0.0167
Child Initiative	13.70 (8.89)	8.33 (6.90)	4.164	17	<0.001	0.786	0.347, 1.225	0.025
Maximal Complexity	3.80 (0.41)	3.33 (0.84)	2.204	17	0.0416 Wilcoxon signed-rank *p* = 0.042	0.65	−0.01, 1.309	0.05

The df change because the complexity and child-initiative variables have two fewer participants—the participants who showed no symbolic play in the triadic condition.

To verify that the exclusion of two children from the complexity and initiative analysis did not bias the results, we re-ran the complexity and initiative comparisons with both children included, coding their triadic values at the floor of each scale (the lowest complexity level and zero child-initiated acts, consistent with an absence of symbolic play). The effects remained significant [maximal complexity: t(19) = 2.77, *p* = 0.012; child initiative: t(19) = 4.47, *p* < 0.001]. The same held in the multivariate test [*N* = 20 for the five measures; Hotelling’s T^2^ = 69.26, *F*(5, 15) = 10.94, *p* < 0.001]. The exclusion was therefore conservative and did not drive the reported differences.

### Exploratory analyses

In addition to examining the research hypotheses, we also examined the difference in overall play duration of toddlers between the conditions (not just the duration of symbolic play). In a paired-samples *t*-test, we found a significant difference in the overall play duration of toddlers between the two conditions, such that the play duration in the dyadic condition (*M* = 213.16, *SD* = 63.53) was longer than the play duration in the triadic condition [*M* = 165.13, *SD* = 68.34, *t*(19) = 3.739, *p* = 0.001, 95%CI (0.284, 1.168), *d* = 0.726].

## Discussion

The aim of the current study was to investigate differences in toddlers’ symbolic play between dyadic interactions, involving only the mother, and triadic interactions involving both the mother and an older sibling. We hypothesized that toddlers’ play would be characterized by a higher symbolic level in the dyadic play interaction compared to the triadic interaction, and that these differences would be reflected in all the measured aspects of play (duration, frequency, initiative, variety, and complexity). This was based on previous research indicating that dyadic play with the mother includes elements that enhance the toddler’s symbolic play (Fiese, 1990), as well as inconsistent findings regarding sibling play (Finn and Vandermaas-Peeler, 2013; Oshima-Takane and Robbins, 2003).

In accordance with our hypothesis, we found that all measures of symbolic play were higher during dyadic play compared to triadic play. In other words, during play with the mother only, compared to play including the older sibling as well, more time was dedicated to symbolic play, more symbolic play episodes occurred, the symbolic play was more varied, had a higher complexity level, and included more toddler-initiated symbolic play.

The current study is the first to conduct a direct and controlled comparison between dyadic and triadic symbolic play interactions. It provides evidence that the advantages observed in dyadic symbolic play also hold true when compared to play in a triadic setting, and not just when compared to toddlers’ solitary play (Fiese, 1990). These findings are particularly important considering that, both at home and in educational settings, it is common for toddlers to interact with other children in the presence of an adult (Netovich, 2024).

A special measure to note is the child-initiative measure, which is neither strictly qualitative nor quantitative. Unlike previous research, which primarily used this measure to learn about maternal involvement in interactions, in the current study, toddler initiatives of symbolic play served as a measure of their play quality. We found that, in dyadic interactions, the quantity of symbolic play initiatives by the toddler was higher compared to triadic interaction. This finding may suggest that, in triadic play, the toddler had fewer opportunities to initiate symbolic play independently and is perhaps less active and independent compared to dyadic play.

Another measure to note is complexity. While the difference between conditions was significant when examining maximal complexity, this measure showed the weakest effect, and the sample size might not have been large enough to detect an effect. This could be because the majority of toddlers showed the same maximal complexity score (which was a score of 4 out of 5). Fourteen out of 18 toddlers in the dyadic condition reached this score, as did nine toddlers in the triadic condition. Because this was also the measure with the lowest inter-judge reliability, to measure toddlers’ complexity of play, perhaps a better coding scheme is necessary. Alternatively, one could test younger toddlers whose play level is naturally less advanced and will show more variability.

The idea that triadic interaction may pose challenges for toddler play is supported by previous evidence suggesting that, when their older siblings are present, toddlers tend to engage in less active participation in interaction compared to when they spend time with their mothers only (e.g., Oshima-Takane and Robbins, 2003). It was also found that, among children who participated in a triadic interaction, parents’ success in spontaneously teaching the younger child was lower, the learning was less adapted to the child’s level, and the interaction included more frustration and conflict, compared to children who experienced a dyadic interaction (e.g., Finn and Vandermaas-Peeler, 2013). Thus, the current study can support both the idea that maternal dyadic interaction fosters symbolic play and the idea that shared interaction with a sibling and a parent may hinder the toddler’s play.

Regarding the relative disadvantage of a triadic compared to a dyadic interaction, no measures were coded to assess the quality of the interaction. However, from our impression during coding, in these situations, mothers had more difficulty maintaining joint play. Additionally, triadic play tended to include more distractions and conflict. Because, in order to provide the toddler favorable conditions for symbolic play, the parent needs to be attentive, responsive, and adapted to the toddler’s level of play (Marjanovič-Umek et al., 2014; Palacios and Rodríguez, 2015; see also Stuart et al., 2023, for evidence linking maternal sensitivity to children’s pretend-play complexity), the challenges outlined above might make it difficult for the parent to meet the conditions required. Future studies may want to systematically code for maternal attention to the younger sibling, or whether siblings are playing together or in parallel.

That being said, we cannot determine whether the advantages of the dyadic condition over the triadic condition necessarily indicate benefit and contribution of parents to play, or whether the comparative findings indicate a potential disadvantage resulting from the presence of older siblings in triadic play (or both). Because there is no objective baseline for the toddler’s symbolic play, this question remains open. Furthermore, because this study did not examine dyadic play of the toddler with their older sibling (without the parent present), we cannot determine whether the apparent disadvantages in triadic play stem from characteristics of the older sibling, or whether they reflect any triadic composition. Thus, the demand on the parent to divide attention between two children and to provide favorable conditions for both simultaneously would also appear in an interaction with an additional adult.

The current study examined specific measures related to symbolic play but did not examine other measures that may benefit from triadic play. For example, during shared play with siblings, exposure to diverse content and additional situations, emotional scenarios, complex social situations, and coping with them, as well as different conversational styles may occur (Barton and Tomasello, 1991; Woollett, 1986). The toddler may also indirectly benefit from being exposed to a richer environment and witnessing exchanges of content and more complex learning (Barton and Tomasello, 1991; Finn and Vandermaas-Peeler, 2013). Moreover, the findings of the current study may support Dunn (1983) suggestion that play with parents and play with siblings could fulfill different roles. While play with parents serves the development of new skills, play with siblings may provide an opportunity for integration and practice of skills that have already been established. Therefore, we cannot conclude that triadic play interactions harm or do not benefit the toddler at all. It seems that the toddler’s experience in different compositions of play in everyday life, including parents, siblings, friends, and other adults, is important. Additionally, it may be important to reserve one-on-one playtime between the parent and child, beyond shared family play.

The current study had several limitations. First, the sample was small and consisted of 20 participants and their families., While this sample size was determined by practical considerations rather than an *a priori* power analysis, several features of the results give us confidence in the main findings: results remained significant after Holm–Bonferroni correction for multiple comparisons, and were associated with high statistical power; and a sensitivity analysis indicated that the design could detect within-subject effects of dz. ≈ 0.66 with 80% power. At the same time, the small sample limits what can be concluded for the weaker effects. In particular, the complexity effect was the smallest, and was the least reliably coded; it should therefore be regarded as preliminary and in need of replication with a larger sample and a more sensitive measure of complexity. More broadly, research on symbolic play has frequently been underpowered (Quinn et al., 2018), and the present findings would benefit from replication in a larger and more diverse sample.

Second, despite the attempt to allow the participants a natural environment as much as possible, by conducting play interactions at the participants’ homes, with toys familiar to them and without the researcher visible, the request to engage in shared play in different family compositions and for 10 consecutive minutes each in front of the camera may be unnatural for some participants. Using Zoom rather than a lab visit, and eliminating the researchers’ presence in the home may have helped mitigate this concern; however, coping with the unnatural situation may be significantly different between one condition and the other. For example, playing with the mother alone for an extended period of time may be a less natural situation for some participants, as they are not accustomed to it from their daily lives, unlike family play. We do not know which play interaction tends to be more common, and how much time is naturally devoted to each type of interaction in toddlers’ daily lives.

A further consequence of testing children in their own homes is that the play materials were not standardized across families, as each toddler played with their own toys; this choice increases ecological validity but introduces variability in the materials available. Importantly, because the design was within-participant, with each toddler playing in both conditions with the same set of toys, differences in toys cannot account for the observed dyadic–triadic differences. To limit other home-based sources of disturbance, families were asked to set up in a quiet room, and the experimenter remained present on the call (with the camera off) throughout each session and was therefore able to note any interruptions or distractions (which did not occur).

Third, the study included only mothers, so it remains open whether the advantage of dyadic play generalizes to father–toddler interactions, particularly given evidence that fathers’ play tends to be more physical and less oriented toward pretend or symbolic play than mothers’ (Amodia-Bidakowska et al., 2020); future work should examine dyadic and triadic configurations that include fathers.

Finally, while significant differences in the symbolic play of toddlers emerged between the two types of play interactions, it was not possible to examine the mechanisms through which this difference occurs. Further research could find a way to examine putative factors influencing children’s symbolic play in triads and dyads.

Relatedly, the present study did not measure individual child characteristics that may shape symbolic play and its sensitivity to the social context. There are substantial individual differences in the amount and sophistication of young children’s social pretend play, linked to the quality of their relationships with mothers and siblings (Youngblade and Dunn, 1995), and children’s own emotional functioning as well as parental psychopathology have been associated with reduced engagement in pretend play (Rao et al., 2021). Factors such as, emotion regulation, language level, or clinical conditions were not assessed here and represent an important direction for future work.

To conclude, the current study examined the differences in the level of symbolic play between dyadic interactions of toddlers with their mothers and triadic interactions that also included an older sibling. Clear differences were found between the two conditions, with all measures of symbolic play considered higher in the dyadic condition compared to the triadic condition. The current study contributes to the limited knowledge about symbolic play in a triadic composition. It can be concluded that mother–child dyadic play has a unique importance. Therefore, at the practical level, it is important for parents to dedicate time to dyadic symbolic play with their children. The study is unable to disentangle the underlying factors behind the findings regarding the relative disadvantage of triadic symbolic play compared to dyadic play, and it is important to continue to investigate the mechanisms that may broaden our understanding of symbolic play in its various family compositions.

## Data Availability

The datasets presented in this study can be found in online repositories. The names of the repository/repositories and accession number(s) can be found at: https://osf.io/rqs8w/overview?view_only=8ea622ebae3b49c0849a2fd2e316c419.

## References

[ref1] Amodia-Bidakowska A. Laverty C. Ramchandani P. G. (2020). Father-child play: a systematic review of its frequency, characteristics and potential impact on children’s development. Dev. Rev. 57:100924. doi: 10.1016/j.dr.2020.100924

[ref2] Baron-Cohen S. (1987). Autism and symbolic play. Br. J. Dev. Psychol. 5, 139–148. doi: 10.1111/j.2044-835X.1987.tb01049.x

[ref3] Barton M. E. Tomasello M. (1991). Joint attention and conversation in mother-infant-sibling triads. Child Dev. 62, 517–529. doi: 10.1111/j.1467-8624.1991.tb01548.x

[ref4] Baskett L. M. Johnson S. M. (1982). The young child's interactions with parents versus siblings: a behavioral analysis. Child Dev. 53, 643–650. doi: 10.2307/1129375

[ref5] Benigno J. P. Clark L. Farrar M. J. (2007). Three is not always a crowd: contexts of joint attention and language. J. Child Lang. 34, 175–187. doi: 10.1017/S0305000906007732, 17340943

[ref6] Benigno J. P. Ellis S. (2004). Two is greater than three: effects of older siblings on parental support of preschoolers’ counting in middle-income families. Early Child Res. Q. 19, 4–20. doi: 10.1016/j.ecresq.2004.01.006

[ref7] Blake J. (1981). Family size and the quality of children. Demography 18, 421–442. doi: 10.2307/2060941 7308532

[ref8] Bronfenbrenner U. (1977). Toward an experimental ecology of human development. Am. Psychol. 32, 513–531. doi: 10.1037/0003-066X.32.7.513

[ref9] Bronfenbrenner U. (1999). “Environments in developmental perspective: theoretical and operational models,” in Measuring Environment across the life span: Emerging Methods and Concepts, eds. Friedman S. L. Wachs T. D. (American Psychological Association), 3–28. doi: 10.1037/10317-001

[ref10] Casby M. W. (2003). The development of play in infants, toddlers, and young children. Commun. Disord. Q. 24, 163–174. doi: 10.1177/15257401030240040201

[ref11] Champely S. (2020) PWR: basic functions for power analysis (R package version 1.3-0) [computer software]. Available online at: https://CRAN.R-project.org/package=pwr

[ref12] Christensen L. Hutman T. Rozga A. Young G. S. Ozonoff S. Rogers S. J. . (2010). Play and developmental outcomes in infant siblings of children with autism. J. Autism Dev. Disord. 40, 946–957. doi: 10.1007/s10803-010-0941-y, 20112084 PMC2904459

[ref13] Colwell M. Lindsey E. (2003). Teacher-child interactions and preschool children's perceptions of self and peers. Early Child Dev. Care 173, 249–258. doi: 10.1080/03004430303096

[ref14] Creaghe N. Kidd E. (2022). Symbolic play as a zone of proximal development: an analysis of informational exchange. Soc. Dev. 31, 1138–1156. doi: 10.1111/sode.12592

[ref15] Creaghe N. Quinn S. Kidd E. (2021). Symbolic play provides a fertile context for language development. Infancy 26, 980–1010. doi: 10.1111/infa.12422, 34297890

[ref16] Downey D. B. (2001). Number of siblings and intellectual development: the resource dilution explanation. Am. Psychol. 56, 497–504. doi: 10.1037/0003-066X.56.6-7.497, 11413873

[ref17] Dunifon R. Fomby P. Musick K. (2017). Siblings and children’s time use in the United States. Demogr. Res. 37, 1611–1624. doi: 10.4054/demres.2017.37.49

[ref18] Dunn J. (1983). Sibling relationships in early childhood. Child Dev. 54, 787–811. doi: 10.2307/1129886

[ref19] Dunn J. Dale N. (1984). “I a daddy: 2-year-olds' collaboration in joint pretend with sibling and with mother,” in Symbolic Play, ed. Bretherton I. (Academic Press), 131–158. doi: 10.1016/b978-0-12-132680-7.50009-0

[ref20] Dunn J. Wooding C. Hermann J. (1977). Mothers' speech to young children: variation in context. Developmental Medicine Child Neurology 19, 629–638. doi: 10.1111/j.1469-8749.1977.tb07996.x, 913903

[ref21] Erikson E. H. (1951). Sex differences in the play configurations of preadolescents. Am. J. Orthopsychiatry 21, 667–692. doi: 10.1111/j.1939-0025.1951.tb00021.x, 14885381

[ref22] Farver J. A. M. Wimbarti S. (1995). Indonesian children's play with their mothers and older siblings. Child Dev. 66, 1493–1503. doi: 10.1111/j.1467-8624.1995.tb00947.x

[ref23] Fein G. G. Fryer M. G. (1995). Maternal contributions to early symbolic play competence. Dev. Rev. 15, 367–381. doi: 10.1006/drev.1995.1014

[ref24] Fiese B. H. (1990). Playful relationships: a contextual analysis of mother-toddler interaction and symbolic play. Child Dev. 61, 1648–1656. doi: 10.1111/j.1467-8624.1990.tb02891.x 1700947

[ref25] Finn L. Vandermaas-Peeler M. (2013). Young children's engagement and learning opportunities in a cooking activity with parents and older siblings. Early Childhood Research Practice 15, 372–383. doi: 10.1080/09669760.2012.663237

[ref26] Fletcher K. Mendelsohn A. Brockmeir C. Tamis-LeMonda C. S. (2020). Play in Mexican- American mothers and toddlers is frequent, multimodal, and rich in symbolic content. Infancy 25, 535–551. doi: 10.1111/infa.12344, 32857437

[ref27] Gamer M. Lemon J. Singh I. F. P. (2019) Irr: various coefficients of interrater reliability and agreement (R package version 0.84.1) [computer software]. Available online at: https://CRAN.R-project.org/package=irr

[ref28] Gurgand L. Lamarque L. Havron N. Bernard J. Y. Ramus F. Peyre H. (2023). The influence of sibship composition on language development at 2 years of age in the ELFE birth cohort study. Dev. Sci. 26:e13356. doi: 10.1111/desc.13356, 36437698

[ref29] Havron N. Lovcevic I. Kee M. Z. Chen H. Chong Y. S. Daniel M. . (2022). The effect of older sibling, postnatal maternal stress, and household factors on language development in two-to four-year-old children. Dev. Psychol. 58:2096. doi: 10.1037/dev0001417 35951397

[ref30] Havron N. Ramus F. Heude B. Forhan A. Cristia A. Peyre H. . (2019). The effect of older siblings on language development as a function of age difference and sex. Psychol. Sci. 30, 1333–1343. doi: 10.1177/0956797619861436

[ref31] Keren M. Feldman R. Namdari-Weinbaum I. Spitzer S. Tyano S. (2005). Relations between parents' interactive style in dyadic and triadic play and toddlers' symbolic capacity. Am. J. Orthopsychiatry 75, 599–607. doi: 10.1037/0002-9432.75.4.599, 16262517

[ref32] Kowalski H. S. (2000) Toddlers' Emerging symbolic play: the Influence of peers in the day-care context. [Master's thesis, University of Wollongong

[ref33] Kowalski H. S. Wyver S. R. Masselos G. De Lacey P. (2004). Toddlers’ emerging symbolic play: a first-born advantage? Early Child Dev. Care 174, 389–400. doi: 10.1080/0300443032000153435

[ref34] Leslie A. M. (1987). Pretense and representation: the origins of" theory of mind.". Psychol. Rev. 94, 412–426. doi: 10.1037/0033-295X.94.4.412

[ref35] Linder T. W. (1993). Transdisciplinary play-based Assessment: A Functional Approach to Working with young children. Rev. Edn Paul H Brookes Publishing. Paul H. Brookes Publishing Co., Baltimore, MD. doi: 10.1177/105381519401800307.

[ref36] Lockman J. J. Tamis-LeMonda C. S. (2021). Young children’s interactions with objects: play as practice and practice as play. Annual Review of Developmental Psychology 3, 165–186. doi: 10.1146/annurev-devpsych-050720-102538, 37859666 PMC10586717

[ref37] Loukatou G. Scaff C. Demuth K. Cristia A. Havron N. (2022). Child-directed and overheard input from different speakers in two distinct cultures. J. Child Lang. 49, 1173–1192. doi: 10.1017/S0305000921000623, 34663486

[ref38] Lyytinen P. Poikkeus A. M. Laakso M. L. (1997). Language and symbolic play in toddlers. Int. J. Behav. Dev. 21, 289–302. doi: 10.1080/016502597384875

[ref40] Marcu I. Oppenheim D. Koren-Karie N. Dolev S. Yirmiya N. (2009). Attachment and symbolic play in preschoolers with autism spectrum disorders. J. Autism Dev. Disord. 39, 1321–1328. doi: 10.1007/s10803-009-0747-y, 19421849

[ref41] Marjanovič-Umek L. Fekonja-Peklaj U. Podlesek A. (2014). The effect of parental involvement and encouragement on preschool children's symbolic play. Early Child Dev. Care 184, 855–868. doi: 10.1080/03004430.2013.820726

[ref42] Netovich T. (2024). The Relationship between Parental Dyadic and Triadic Interactions and Sibling Language Development (Master's thesis). Haifa: University of Haifa. [in Hebrew].

[ref43] O'connell Bretherton I. (1984). “Toddler's play, alone and with mother: the role of maternal guidance,” in Symbolic Play, ed. Bretherton I. (Orlando, FL: Academic Press), 337–368. doi: 10.1016/B978-0-12-132680-7.50016-8

[ref44] Oden S. Hall J. A. (2021). “Peer and sibling influences on play,” in Play from Birth to Twelve and beyond, Routledge, New York, 266–276. doi: 10.4324/9781003249702-40

[ref45] Oshima-Takane Y. Robbins M. (2003). Linguistic environment of secondborn children. First Lang. 23, 21–40. doi: 10.1177/0142723703023001002

[ref46] Palacios P. Rodríguez C. (2015). The development of symbolic uses of objects in infants in a triadic context: a pragmatic and semiotic perspective. Infant Child Dev. 24, 23–43. doi: 10.1002/icd.1873

[ref47] Pedersen T. L. (2025) Patchwork: the composer of plots (R package version 1.3.2) [computer software]. Available online at: https://CRAN.R-project.org/package=patchwork

[ref48] Piaget J. (1962). The relation of affectivity to intelligence in the mental developmentof the child. Bull. Menn. Clin. 26:129. 14486279

[ref49] Quinn S. Donnelly S. Kidd E. (2018). The relationship between symbolic play and language acquisition: a meta-analytic review. Dev. Rev. 49, 121–135. doi: 10.1016/j.dr.2018.05.005

[ref9001] R Core Team (2020). R: A Language and Environment for Statistical Computing. Vienna: R Foundation for Statistical Computing. Available online at: https://www.R-project.org/

[ref50] Rao Z. Barker B. O’Farrelly C. Ramchandani P. (2021). Maternal anxiety and depression and their associations with mother–child pretend play: a longitudinal observational study. BMC Psychol. 9:70. doi: 10.1186/s40359-021-00568-9, 33957981 PMC8103647

[ref51] Rogoff B. (2003). The Cultural Nature of human Development. New York: Oxford University Press.

[ref52] Rosslund A. Kartushina N. Serres N. Mayor J. (2025). Early vocabulary acquisition: From birth order effect to child-to-caregiver ratio. Child Development, 96, 1343–1353. doi: 10.31234/osf.io/nk4g7 40317493

[ref53] Rutherford M. D. Rogers S. J. (2003). Cognitive underpinnings of pretend play in autism. J. Autism Dev. Disord. 33, 289–302. doi: 10.1023/A:1024406601334, 12908832

[ref54] Signorell A. (2025) DescTools: tools for descriptive statistics (R package version 0.99.60) [computer software]. Available online at: https://CRAN.R-project.org/package=DescTools

[ref55] Slade A. (1987). A longitudinal study of maternal involvement and symbolic play during the toddler period. Child Dev. 58, 367–375. doi: 10.2307/1130513 2435464

[ref57] Stevenson M. B. Leavitt L. A. Thompson R. H. Roach M. A. (1988). A social relations model analysis of parent and child play. Dev. Psychol. 24, 101–108. doi: 10.1037/0012-1649.24.1.101

[ref58] Stuart A. C. Gufler S. R. Tharner A. Væver M. S. (2023). Story stems in early mother-infant interaction promote pretend play at 30 months. Infant Behav. Dev. 74:101893. doi: 10.1016/j.infbeh.2023.101893, 37844456

[ref59] Stuart A. C. Roos C. O. Smith-Nielsen J. Egmose I. Væver M. S. (2023). Four-year-old children’s pretend play complexity during free play and story stem play and associations with maternal sensitivity. Scand. J. Psychol. 64, 644–651. doi: 10.1111/sjop.12920, 37035921

[ref60] Teti D. M. Bond L. A. Gibbs E. D. (1988). Mothers, fathers, and siblings: a comparison of play styles and their influence upon infant cognitive level. Int. J. Behav. Dev. 11, 415–432. doi: 10.1177/016502548801100402

[ref61] Torchiano M. (2020). Effsize: efficient effect size computation (R package version 0.8.1) [computer software]. doi: 10.5281/zenodo.1480624

[ref62] Ungerer J. A. Sigman M. (1981). Symbolic play and language comprehension in autistic children. J. Am. Acad. Child Psychiatry 20, 318–337. doi: 10.1016/S0002-7138(09)60992-4, 6167603

[ref63] Vygotsky L. S. (1967). Play and its role in the mental development of the child. Sov. Psychol. 5, 6–18. doi: 10.2753/RPO1061-040505036

[ref64] Vygotsky L. S. (1987). The Collected Works of LS Vygotsky: Problems of the Theory and History of Psychology, vol. 3. Plenum Press, New York: Springer Science & Business Media.

[ref65] Wickham H. (2016). ggplot2: Elegant Graphics for data Analysis. New York: Springer-Verlag.

[ref66] Wittenburg P. Brugman H. Russel A. Klassmann A. Sloetjes H. (2006). “ELAN: a professional framework for multimodality research,” in Proceedings of LREC 2006, Fifth International Conference on Language Resources and Evaluation, (Genoa, Italy: European Language Resources Association (ELRA)).

[ref9003] Woollett A. (1986). The influence of older siblings on the language environment of young children. British Journal of Developmental Psychology 4, 235–245. doi: 10.1111/j.2044-835X.1986.tb01015.x

[ref67] Youngblade L. M. Dunn J. (1995). Individual differences in young children's pretend play with mother and sibling: links to relationships and understanding of other people's feelings and beliefs. Child Dev. 66, 1472–1492. doi: 10.1111/j.1467-8624.1995.tb00946.x, 7555225

